# MiR-125b Suppression Inhibits Apoptosis and Negatively Regulates Sema4D in Avian Leukosis Virus-Transformed Cells

**DOI:** 10.3390/v11080728

**Published:** 2019-08-07

**Authors:** Chaoqi Ren, Ruyu Xie, Yongxiu Yao, Mengmeng Yu, Fangfang Chang, Lixiao Xing, Yao Zhang, Yongzhen Liu, Suyan Wang, Muhammad Farooque, Yongqiang Wang, Xiaole Qi, Changjun Liu, Yanping Zhang, Hongyu Cui, Kai Li, Li Gao, Qing Pan, Venugopal Nair, Xiaomei Wang, Yulong Gao

**Affiliations:** 1 Division of Avian Infectious Diseases, State Key Laboratory of Veterinary Biotechnology, Harbin Veterinary Research Institute, The Chinese Academy of Agricultural Sciences, Harbin 150069, China; 2 Department of Life science, Northeast Forestry Universtiy, Harbin 150069, China; 3 The Pirbright Institute & UK-China Centre of Excellence for Research on Avian Diseases, Pirbright, Ash Road, Guildford, Surrey GU24 0NF, UK

**Keywords:** miR-125b, Subgroup J avian leukosis virus, Sema4D, cell apoptosis

## Abstract

Subgroup J avian leukosis virus (ALV-J), an oncogenic retrovirus, causes hemangiomas and myeloid tumors in chickens. We previously showed that miR-125b is down-regulated in ALV-J-induced tumors. This study aimed to investigate the possible role of miR-125b in ALV-J-mediated infection and tumorigenesis. Knockdown of miR-125b expression in HP45 cells reduced, whereas over-expression induced late-stage apoptosis. Bioinformatics analysis and luciferase activity assays indicate that miR-125b targets Semaphorin 4D/CD100 (Sema4D) by binding the 3′-untranslated region of messenger RNA (mRNA). Up-regulation of miR-125b in the DF1 cell line suppressed Sema4D expression, whereas miR-125 down-regulation increased Sema4D expression levels. To uncover the function of Sema4D during ALV-J infection, animal infection experiments and in vitro assays were performed and show that *Sema4D* mRNA levels were up-regulated in ALV-J-infected tissues and cells. Finally, functional experiments show that miR-125 down-regulation and Sema4D over-expression inhibited apoptosis in HP45 cells. These results suggest that miR-125b and its target Sema4D might play an important role in the aggressive growth of HP45 cells induced by avian leukosis viruses (ALVs). These findings improve our understanding of the underlying mechanism of ALV-J infection and tumorigenesis.

## 1. Introduction

Avian leukosis viruses (ALVs) are type C retroviruses that are associated with various neoplasms such as lymphoid and myeloid leukosis in several organs [1]. ALV can be divided into the exogenous subgroups A, B, C, D, J, and K, and the endogenous subgroup E based on cross-neutralization patterns, viral envelope glycoprotein properties, and host range in chickens [2,3]. Compared to other ALV subgroups, the exogenous subgroup J (ALV-J) has a broad host range and enhanced pathogenicity. ALV-J mainly induces myelocytic myeloid leukosis (myelocytomatosis) [2]. Since the first report of myeloid leukosis (ML) in Britain caused by ALV-J, the virus has resulted in increasingly severe damage to the poultry industry worldwide. In China, ALV-J was first isolated in 1999 [4]; subsequently, it became widespread through horizontal and vertical transmission, causing increased economic losses owing to severe immunosuppression and tumor-related mortality [5,6]. Increasing evidence suggesting that some viruses induce tumor formation via multifunctional oncogenes present in their own genomes [7]. However, ALV-J does not express a viral oncogene and induces multiple tumors, possibly through alternative mechanisms. The integration of ALV-J into the host genome can lead to the activation of proto-oncogenes or the inactivation of tumor suppressors in the host to induce tumors [8,9,10]. Therefore, other mechanisms might be associated with ALV-J infection and tumorigenicity.

MicroRNAs (miRNAs) are endogenous, single-stranded, noncoding RNAs of approximately 22 nucleotides in length. Hundreds of miRNAs have been reported to play an important role in virus infection and tumorigenesis. To date, 674 miRNAs have been identified in chickens using the miRBase database, many of which have been identified as oncogenes or tumor suppressors in different human cancers [11,12]. MiR-155, the first microRNA shown to be activated by ALV, has also been found to suppress apoptosis in chicken tumors [13,14]. Moreover, the dysregulation of oncogene-associated miRNAs is a key mediator of cellular function during tumor formation [13]. Several dysregulated miRNAs (e.g., miR-21, miR-101, miR-195, miR-125b, and miR-224) have been shown to regulate cell growth, apoptosis, migration, and invasion [14,15,16,17,18]. These findings indicate that miRNA dysregulation is associated with tumorigenesis. Among the abnormally expressed miRNAs, down-regulation of host-encoded miRNAs in response to oncogenic viral infection might be a common feature of tumor formation [19,20]. We previously demonstrated the down-regulation of miR-125b by approximately two fold in ALV-J-induced liver tumors [21]. Other reports have shown that down-regulation of miR-125b expression is associated with several human cancers [22]. However, the mechanism through which miR-125b down-regulation during ALV-J infection in chickens underlies tumor formation has not been well studied.

The verification of host-dysregulated miR-125b and the exploration of its functional mechanisms might provide a better understanding of ALV-J-mediated tumorigenesis, resulting in improved strategies to control the pathological effects of this virus. In this study, we first tested the function of miR-125b in apoptosis, and then identified Semaphorin 4D/CD100 (Sema4D) as a miR-125b-target in chickens; further, we determined the potential function of miR-125b and Sema4D in apoptosis.

## 2. Materials and Methods

### 2.1. Cells and Viruses

The human embryonic kidney (HEK) 293T cell line and chicken DF1 cells, a continuous chicken embryo fibroblast cell line, were obtained from the American Type Culture Collection (ATCC). The HP45 cell line was isolated from chicken bursa lymphoma induced by the ALV HPRS F42 strain by the Regional Poultry Research Laboratory (East Lansing, MI, USA) [23,24]. HP45 cells were used as an in vitro model of ALV infection in this study. 293T and DF1 cell lines were cultured in Dulbecco’s modified Eagle’s medium (DMEM; Thermo Scientific, Rockford, IL, USA), the HP45 cells were cultured in Roswell Park Memorial Institute (RPMI) 1640 medium (GIBCO, high glucose 4.5 g/L). Both DMEM and RPMI1640 contained 10% fetal bovine serum (FBS; Sigma-Aldrich, St. Louis, MO, USA), 1000 U/L penicillin-streptomycin (Invitrogen, Carlsbad, CA, USA), and 1% sodium pyruvate. RPMI 1640 also contain 10% tryptose phosphate broth and 0.1% 2-mercaptoethanol. All cell lines were cultured in a humidified incubator with an atmosphere of 5% CO_2_ at 37 °C. The HLJ09SH01 (GenBank HQ634806) strain used in this study was propagated in DF-1 cells, and conserved by our laboratory, which was isolated from a commercial layer chicken [25].

### 2.2. Plasmid Construction

The miR-125b-5p mimic, inhibitor, and negative controls (NCs) were synthesized by TianGen (Beijing, China). The 3′-untranslated region (3′-UTR) of *Sema4D* (GenBank No.: 396331) containing predicted miR-125b seed sequences (5′-CUCAGGG-3′), termed pMir-Sema4D-WT, or containing mutated seed sequences (5′-UGUCAAA-3′; ~300 bp), termed pMir-Sema4D-MT, were synthesized by GenScript Biotech (Nanjing, China). Sema4D-WT and Sema4D-MT 3′-UTR were cloned downstream of the *Renilla* luciferase reporter gene of the pMir-GLO vector (pMir-GLO; Promega, Fitchburg, WI, USA) with 5′-*NheI* and 3′-*SbfI* sites to generate pMir-Sema4D-WT and pMir-Sema4D-MT plasmids. 

The Sema4D fragment was obtained from chicken DF1 cell genomic cDNA with specific primers for chicken Sema4D as follows: forward 1, 5′-GATTACGCTGAATTCATGACTCTGCTTGCTTTT-3′; reverse 1, 5′-ATAATATTGAAAATTAAATTC-3′, forward 2, 5′-GAATTTAATTTTCAATATTAT-3′; reverse 2, 5′-AGCTCTGCTGTGCTCCCATGC-3′, forward 3, 5′-GCATGGGAGCACAGCAGAGCT-3′; reverse 3, 5′-AGATCTGCTAGCTCGAGTCAGTCTCCTTCCAC-3′. Then, we linked these three fragments by fusion PCR, and cloned *Sema4D* cDNA into the *XhoI*/*EcoRI* sites of the pCAAGS-HA vector (pCAH) harboring an N-terminal HA tag to generate the pCAH-Sema4D plasmid and pCAH-Blank plasmid as a negative control.

### 2.3. Apoptosis Assays

HP45 cells were cultured in 6-well plates (at 80% confluence) and were transfected with the miR-125b mimic (50 nM) or negative control at 50 nM, the miR-125b inhibitor (100 nM) or negative control at 100 nM, using Lipofectamine RNAiMAX (ThermoFisher, Carlsbad, CA, USA), and pCAH-Sema4D (2 μg) or pCAH-Blank (2 μg) plasmids using X-tremeGENE DNA Transfection Reagent (6 μL) (Roche, Indianapolis, IN, USA). All transfection regents were used according to the manufacturers’ protocols. At 48 h after transfection, the HP45 cells were digested with trypsin and washed twice with phosphate-buffered saline (PBS, pH 7.4). Cells were resuspended in 100 μL of 10× Annexin V binding buffer; then, 5 μL of Annexin V was added and the tubes were incubated in the dark for 15 min at room temperature. Next, 5 μL of propidium iodide (PI) was added to each reaction tube, which was incubated in the dark for 15 min at room temperature; 400 μL of 10× Annexin V binding buffer was added to each reaction tube (Annexin V: FITC Apoptosis Detection Kit, USA). The fluorescence was tested by flow cytometry (Beckman Coulter, Fullerton, CA, USA). Three independent experiments were performed. The rate of apoptosis was analyzed using FlowJo 7.6.1 software and Graphpad prism 6 software.

### 2.4. Small RNA Extraction and Quantification

Total small RNA was extracted from DF1 cells with the mirVana™ miRNA Isolation Kit (Thermo Fisher, USA). The cDNA was synthesized with the miRNA First-strand cDNA Synthesis Kit (Agilent Technologies, CA, USA) with universal RT primers. Real-time PCR (qPCR) was performed with the Applied Biosystems™ qPCR system (Thermo Fisher, USA) using gga-miR-125b specific primers purchased from Tiangen (code: CD202-0045) and the miRcute Plus miRNA qPCR Detection Kit (Tiangen, Beijing, China) following the manufacturer’s instructions. Chicken 5S rRNA was used as an endogenous control for miR-125b expression. The relative expression level of each gene was analyzed using the 2 −∆∆Ct method. 

### 2.5. RNA Extraction and Quantification

Total RNA from tissues and DF1 cells was extracted using TRIzol reagent (Biosharp, Hefei, China). One step RT-qPCR was performed using a BioRT Real Time RT-PCR Kit (Bioer, Hangzhou, China) with *β-actin* as an endogenous control for *Sema4D*. RT-qPCR was performed on the Applied Biosystems™ qPCR system (Thermo Fisher, USA) using specific primers for chicken *Sema4D* (forward, 5′-TGGGTACGGTACAATGGGG-3′; reverse, 5′-CTCTTTACAAGGCGGGGTC-3′). The relative expression level of *Sema4D* was calculated using the 2 −∆∆Ct method.

### 2.6. Bioinformatics Analysis

To predict potential miR-125b-5p-binding sites in the 3′-UTR of targets, we carried out computational analyses with the following databases: miRDB (http://www.mirdb. org/miRDB/index.html) and TargetScan (http://www.targetscan.org/). Gene Ontology (GO) (http://www.geneontology.org/) enrichment analysis was performed to predict the potential functions of miR-125b targets in chicken. KEGG (http://www. genome.jp/kegg/) pathway analysis was performed on predicted targets to identify the biological pathways associated with tumorigenesis. 

### 2.7. Dual Luciferase Assays

To investigate whether miR-125b binds the 3′-UTR seed regions of *Sema4D*, dual luciferase assays were performed. 293T cells (96-well plates) were co-transfected with miR-125b mimic (50 nM) and pMir-Sema4D-WT (0.1 μg) (experiment group), miR-125b inhibitor (100 nM) and pMir-Sema4D-WT (0.1 μg) (negative control group), or miR-125b mimic and pMir-Sema4D-MT (blank group) using Lipofectamine RNAiMAX (ThermoFisher, Carlsbad, USA). Firefly and *Renilla* luciferase activities were tested using the Steady-Glo^®^ luciferase assay system (Promega), 48 h after co-transfection, with an illuminometer (PE Envision, PE, USA). The relative expression of *Renilla* luciferase was determined through normalization to background firefly luciferase levels for each sample.

### 2.8. Animal Infection Assay

Specific pathogen-free chickens (SPF, *n* = 42) at 1 day of age were randomly divided into two groups including a control group and an infection group and maintained separately in a negative-pressure isolator. For the infected group, SPF chickens received ALV-J HLJ09SH01 at a 10^3.5^ tissue culture infective dose (TCID_50_) in 0.5 mL. The SPF chickens were inoculated with an equivalent volume of sterilized PBS in the control group. From the beginning of the 1-week infection, the chickens (*n* = 6) were sacrificed and kidney samples were used to test the expression of *Sema4D* by RT-qPCR and testing lasted 7 weeks. The animal experiments with chickens were approved by the Ethical and Animal Welfare Committee of Heilongjiang Province, China.

### 2.9. DF1 Cell Infection Assay

DF1 cells cultured in 6-well plates at 80% confluence were infected with ALV-J strain HLJ09SH01 at a multiplicity of infection (MOI) of 0.1. Moreover, after a 6-h infection, the media containing viruses were exchanged with fresh DMEM. After a 48-h infection, RNA was extracted from DF1 cells, and RT-qPCR was performed to identify the mRNA expression of *Sema4D*.

### 2.10. Statistical Analysis

All experiments were carried out with at least three independent replicates. The data were provided as means ± standard deviation (SD) values. Differences in data were evaluated by performing a Student’s *t* test. Unadjusted *P*-values < 0.05 were considered statistically significant.

## 3. Results

### 3.1. MiR-125b Positively Associated with Apoptosis Rate

**Figure 1:**
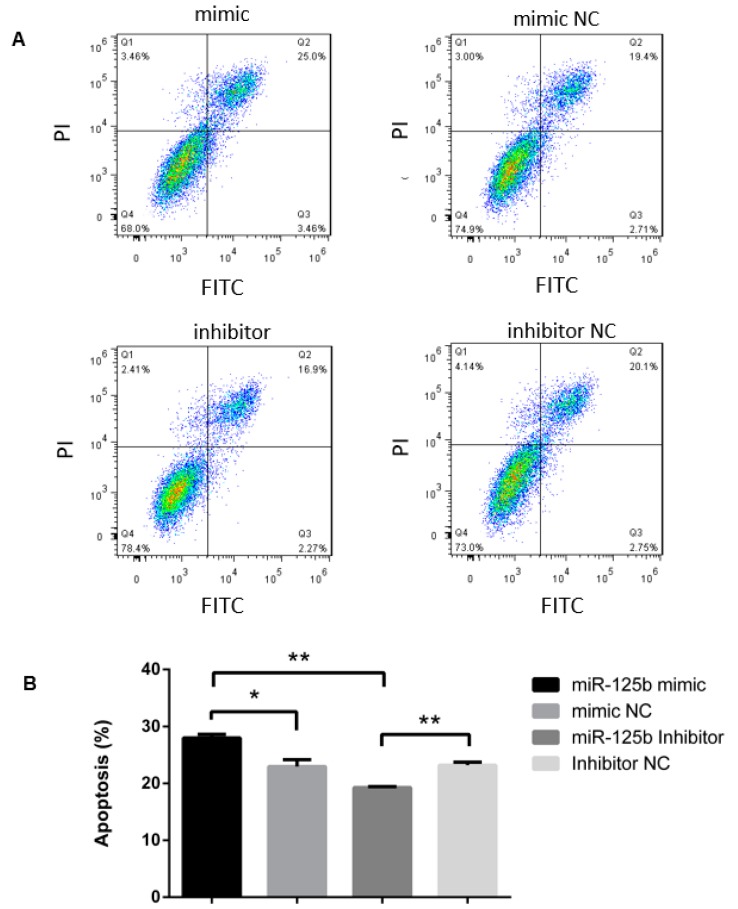
miR-125b is positively related to the apoptosis rate. (**A**) The effect of miR-125b on HP45 cell apoptosis was analyzed by flow cytometry. A miR-125b mimic or mimic negative control (NC; 50 nM) and a miR-125b inhibitor (100 nM) or NC were transfected into HP45 cells in 6-wells plate. Q1 represents cell death; Q2 represents late-stage apoptotic cells; Q3 represents early apoptotic cells; Q4 represents normal cells. (**B**) The flow cytometry results were analyzed by prism software to quantified the effect of miR-125b on apoptosis. Data are provided as the means ± standard deviation for triplicate measurements from a representative experiment. * *p* < 0.05; ** *p* < 0.01.

A previous study in our lab showed that miR-125b is down-regulated in ALV-J-induced tumors [21]. Moreover, tumor development was found to be related to cell proliferation and the dysregulation of apoptosis [26]. MiR-125b, a tumor-associated miRNA in human tumors, has been reported to be associated with apoptosis [27]; however, this process is not well studied with respect to ALV-J-mediated tumorigenesis in chickens. Therefore, we first assessed apoptosis, and the results show that a miR-125b inhibitor suppressed progression to the late stage of apoptosis in HP45 cells. Conversely, miR-125b over-expression promoted HP45 cell apoptosis; this effect was mainly associated with late-stage apoptosis (Q2 areas), whereas early-stage apoptosis (Q3 areas) was virtually unaffected (Figure 1A). Figure 1B shows the significant results from the FACS data. Flow cytometry results were analyzed by prism software to quantify the effects of miR-125b on cell apoptosis (Figure 1B, *p* < 0.05). 

### 3.2. Prediction of miR-125b Target Genes

MiRNAs typically play a very important role in different biological processes by affecting expression of their target genes [11]. To better understand the cellular function of miR-125b during ALV-J infection, we used different databases to predict potential target genes of miR-125b. A total of 191 target genes were predicted using TargetScan and 190 were predicted using miRDB. Twenty-five target genes were common to both lists.

To screen targets associated with apoptosis, we performed biological process, molecular function, and pathway prediction analyses on the 25 common targets using GO enrichment and the KEGG database (Table 1). Sema4D has been well studied with respect to human tumor progression, such as during apoptosis of vaginal epithelial cells [28]. Therefore, we selected Sema4D, which is closely associated with tumor development, to uncover the miRNA–mRNA network and function of ALV-J in tumorigenesis.

### 3.3. MiR-125b Binds to the Sema4D 3′ UTR

**Figure 2:**
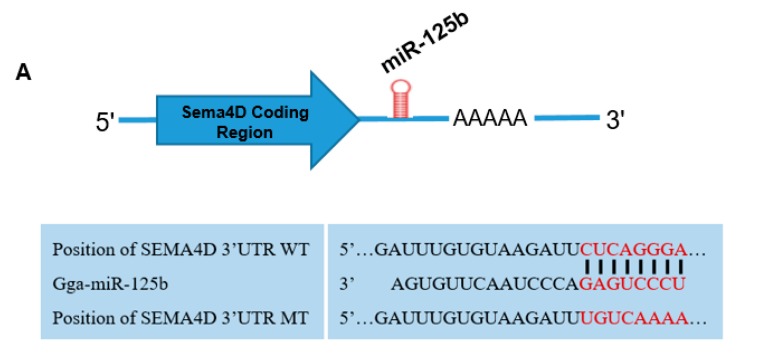

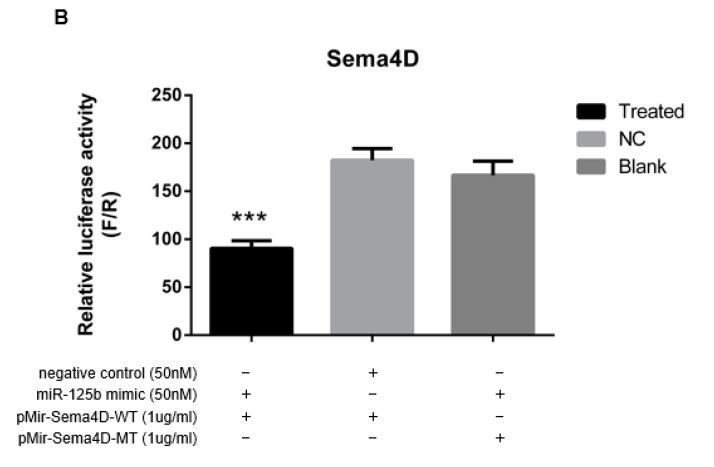
miR-125b binds the Sema4D 3′UTR. (**A**) TargetScan predicted the seed regions of the *Sema4D* 3′-UTR that bind miR-125b. (**B**) Luciferase assays were performed after co-transfecting reporter plasmids with a miR-125b mimic or inhibitor as follows: Treated, miR-125b mimic (50 nM) co-transfected with the pMir-Sema4D-WT (containing the wild type seed sequence) luciferase reporter; NC, miR-125b inhibitor co-transfected with the pMir-Sema4D-WT luciferase reporter; Blank, miR-125b mimic co-transfected with pMir-Sema4D-MT (containing the mutated seed sequence). Luciferase activities were tested 48 h post-transfection. The data are provided as means ± standard deviation for three replicates from a representative experiment. *** *p* < 0.001.

To determine whether *Sema4D* is a target gene of miR-125b in chickens, we predicted the miR-125b-binding seed region in the 3′ UTR of targets using TargetScan. The results show that the 7-nt binding seed regions of miR-125b are perfectly complementary to two 7-nt regions of the *Sema4D* 3′ UTR (Figure 2A).

To further confirm that the seed region of miR-125b binds to the 3′-UTR of *Sema4D*, a luciferase assay was performed. The results show that co-transfection of pMir-Sema4D-WT and the miR-125b mimic decreased luciferase activity (F/R) by 50% (*p* < 0.0001); however, no change in luciferase activity was observed in the NC (miR-125b inhibitor co-transfected with pMir-Sema4D-WT) and blank (miR-125b mimic co-transfected with pMir-Sema4D-MT) groups (Figure 2B). These results demonstrate that miR-125b binds to the 3′ UTR of *Sema4D*.

### 3.4. MiR-125b Suppresses Sema4D Expression

**Figure 3:**
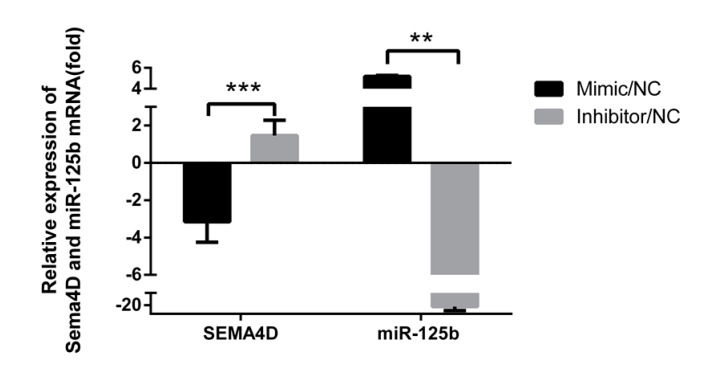
miR-125b suppresses *Sema4D* expression. DF1 cells were transfected with the miR-125b mimic, inhibitor, and negative control (NC). miR-125b and *Sema4D* expression was tested by RT-qPCR 48 h after transfection. Transcript levels of miR-125b and *Sema4D* were normalized to chicken 5S rRNA expression levels. Data are provided as means ± standard deviation values of three replicates from a representative experiment. ** *p* < 0.01; *** *p* < 0.001.

To further verify that miR-125b targets and suppresses *Sema4D*, endogenous *Sema4D* expression in DF1 cells was determined using RT-qPCR. The results show that miR-125b over-expression decreased the mRNA expression of *Sema4D* by approximately 3-fold compared to that in the mimic NC group. Further, knockdown of miR-125b caused an increase in *Sema4D* expression of approximately 2-fold compared to that in the inhibitor NC group (*p* < 0.001; Figure 3). These results clearly indicate that miR-125b targets *Sema4D* and that miR-125b suppresses *Sema4D* mRNA expression in chickens.

### 3.5. ALV-J Up-Regulates Sema4D Expression In Vivo and In Vitro

**Figure 4:**
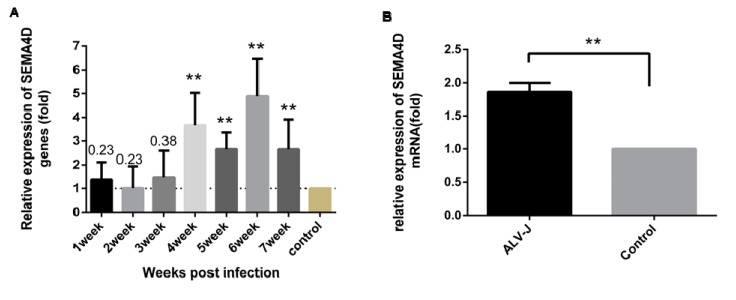
ALV-J up-regulates Sema4D expression in vivo and in vitro. (**A**) Sema4D expression was tested in the kidney tissue of SPF chickens infected with ALV-J by RT-qPCR at different times after infection. (**B**) *Sema4D* expression was tested in DF1 cells infected with ALV-J for 48 h by RT-qPCR. Data are provided as means ± standard deviation values of three replicates from a representative experiment. ** *p* < 0.01.

Studies have shown that Sema4D is related to tumorigenesis, especially with respect to the activation of apoptosis pathways [29]. Moreover, we have verified that Sema4D is a target of miR-125b, an ALV tumorigenesis-associated factor. Thus, to validate the function of Sema4D during ALV-J infection in chickens, we determined the mRNA expression levels of Sema4D in chicken tissues and DF1 cells infected with ALV-J. First, we determined *Sema4D* mRNA expression in chicken tissues from ALV-J-infected SPFs using one-step RT-qPCR. The results show an increase in *Sema4D* mRNA expression, of approximately 3-fold, 4–7 weeks post-ALV-J infection, as compared to that in tissues from normal SPF chickens (Figure 4A; *p* < 0.05). Further, the mRNA expression level of *Sema4D* was increased by 1.8-fold in DF1 cells infected with ALV-J for 48 h (Figure 4B; *p* < 0.001). All these results suggest that ALV-J infection induces *Sema4D* mRNA expression in vivo and in vitro.

### 3.6. Sema4D Suppresses HP45 Cell Apoptosis

**Figure 5:**
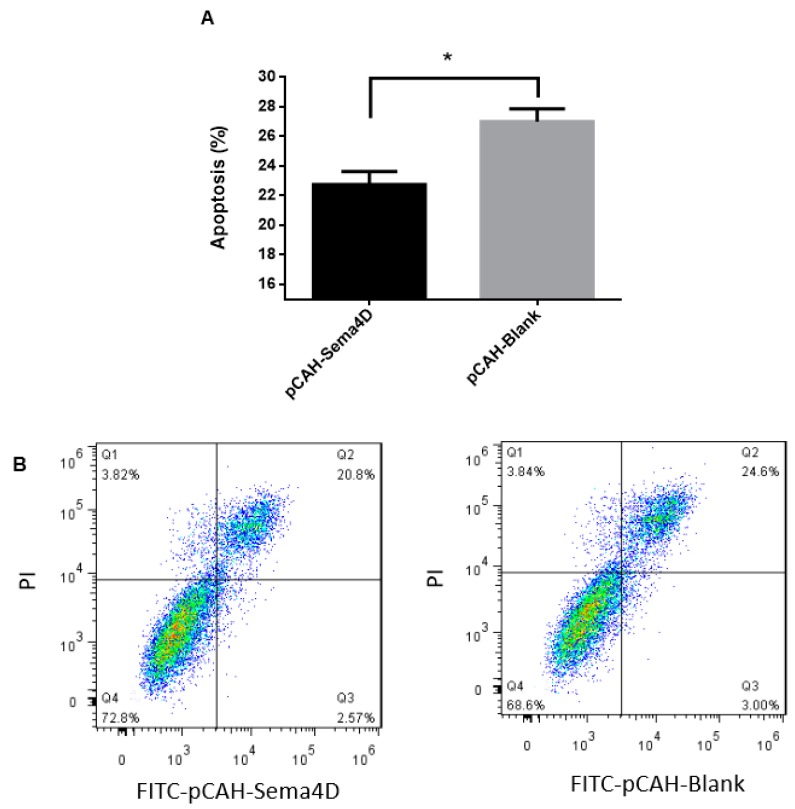
Sema4D suppresses HP45 cell apoptosis. (**A**) pCAH-Sema4D (2 μg) or pCAH-Blank (blank vector) expression vectors were transfected into HP45 cells in 6-well plates. (**B**) Flow cytometric analysis of the effect of miR-125b on HP45 cell apoptosis. Q1 represents dead cells; Q2 represents late-stage apoptotic cells; Q3 represents the early-stage apoptotic cells; Q4 represents normal cells. Data are provided as means ± standard deviation of triplicate measurements from a representative experiment. * *p* < 0.05.

Sema4D has been reported to play an important role in the progression of apoptosis in several human and mouse tumor cells [30,31]. We thus analyzed Sema4D homology among different species. The results show that Sema4D was approximately 68% conserved among chickens and humans and approximately 67% conserved among chickens and mice [32]. Based on the conserved sequence and the promoting role of miR-125b in cell apoptosis, we next determined whether chicken SEMA4D, a target of miR-125b, has the same function in cell apoptosis. Cell apoptosis assays by flow cytometry show that pCAH-Sema4D over-expression reduced the proportion of HP45 cells undergoing late-stage apoptosis as compared to that in the pCAH-Blank group (*p* < 0.05; Figure 5A,B). Therefore, the function of Sema4D overexpression is similar to that of the miR-125b inhibitor with respect to chicken cell apoptosis, which suggests that Sema4D is not only a transcriptional target, but also a functional target, of miR-125b.

## 4. Discussion

ALV-J induces tumorigenesis via various pathways such as ALV-J genome insertion-induced oncogene activation [33]. However, host tumor suppressors also play an inhibitory role in ALV-J tumorigenesis. Abnormally expressed miRNAs might inhibit tumor formation and their down-regulation could be a biomarker of tumorigenesis [34]. A previous study in our lab showed that miR-125b is significantly down-regulated in ALV-J-induced tumors [21]. In the current study, we explored the possible role of miR-125b during ALV-J infection and tumorigenesis. First, we found that a miR-125b inhibitor prevents apoptosis in HP45 cells. Then, bioinformatics approaches and luciferase assays show that *Sema4D* is a target of miR-125b in chickens. Further, we found that ALV-J infection induces the up-regulation of *Sema4D* in vitro and in vivo. Finally, functional experiments show that Sema4D suppresses HP45 cell apoptosis, which is the opposite effect of miR-125b. Identification of the potential miR-125b–Sema4D network during the progression of apoptosis might provide new insights for the understanding of the mechanisms associated with ALV-J infection and tumorigenesis.

ALV-J mainly induces myelocytic myeloid leukosis [2]. Tumor formation occurs owing to unlimited cell proliferation and abnormal apoptosis [35]. Apoptosis, or programmed cell death, is a controlled physiological process in host tissues that functions to remove unwanted cells, including virus-infected cells [36]. To overcome host defenses, many viruses encode anti-apoptotic factors or suppress host apoptotic responses to inhibit this process [37]. Several miRNAs such as miR-21, miR-218, and miR-365 [38,39,40] have been reported to play an important role in aberrant apoptosis, which is a main mechanism of tumor formation. MiR-125b is reportedly associated with human cell apoptosis during the formation of multiple tumors [41,42]. In this study, our results demonstrate that miR-125b plays an important role in chicken HP45 cell apoptosis, mainly during the late stage. Studies have shown that late-stage apoptosis not only functions in tumorigenesis, but also induces more effective activation of the innate immune system [43,44]. ALV-J induces the formation of multiple tumors and immunosuppression in the chicken host [45]. Moreover, an abnormal immune response is an important underlying mechanism of tumor formation [46]. The induction of late-stage apoptosis by miR-125b suggests that it might function as a potential tumor suppressor during ALV-J-induced tumorigenesis.

Both DNA and RNA viruses have evolved mechanisms to degrade, boost, or hijack cellular miRNAs to benefit the viral life cycle [47]. Studies have shown that viruses cause miRNA degradation in a sequence-specific and binding-dependent manner [48]. Retroviruses transcriptionally activate miRNA based on their location at the insert sites [49], and viral proteins trigger the activation of some pathways to influence miRNA expression [50]. In addition, host miRNAs might be essential for the regulation of viral RNA stability, expression, and translation, as well as viral replication and infection [51]. The mechanism underlying the effect of ALV-J on host miR-125b still requires further research.

There are reports that miR-125b is down-regulated and functions as a potential biomarker in osteosarcoma [52]. Conversely, up-regulation of miR-125b is associated with poor prognosis in HER2-positive gastric cancer and in MGMT promoter-unmethylated glioblastoma [53,54]. These dual effects of miR-125b could be attributed to organ-specific actions and diverse cellular contexts in different tumors. The homology of miR-125b among almost all species tested was found to be 100%. In this study, we found that miR-125b promotes apoptosis in HP45 cells, whereas this effect was not significant in DF1 cells [32]. These data suggest that miRNA–mRNA networks might have organ- or cell-specific roles. Whether this miRNA has any other functions in other organs or cells requires further study. A better understanding of the underlying mechanisms of miR-125b and its targets in chickens might provide an overall view of ALV-J tumorigenesis and pathogenicity.

Each miRNA regulates many targets, even when these targets participate in the same cellular process. The identification and characterization of the targets of altered miRNAs might help to elucidate the molecular mechanisms involved in carcinogenesis. MiR-221 can regulate p27 to promote glioblastoma cell proliferation [55], and it can also target LASS2 to promote Schwann cell proliferation and migration [56]. In addition, miR-125b contributes to ovarian granulosa cell apoptosis by targeting BMPR1B [57], and its expression affects the proliferation and apoptosis of human glioma cells by targeting Bmf [41]. All of these reports suggest that miRNAs function by targeting different genes. To explore the mechanisms of miR-125b in ALV-J tumorigenesis, we first screened a dozen potential targets via seed region, GO, and KEGG analyses. These targets included *Sema4D* and other potential targets such as *KLF13*, *PAFAH1B1*, and *PRDM1* [32]. STRING network analysis show that Sema4D interacts with the tumorigenesis-associated protein MET [8], and that it may have roles in multiple processes such as apoptosis during tumor formation and immune functions in chickens. MiR-125b is dysregulated in many human and mouse cancers and in some cell lines, and Sema4D has been reported to be a tumor-associated molecule [58,59,60]. In addition, in humans, Sema4D functions in apoptosis, axonal outgrowth, the nervous system, and the immune system [28,61,62,63]. Our previous study showed that ALV-J down-regulates miR-125b and the present study shows that a miR-125b inhibitor prevents HP45 cell apoptosis. In addition, Sema4D, a target gene of miR-125b, had the same effect on the progression of apoptosis. Apoptosis is very important to counter-balance the cell-generating effects of mitosis. When the apoptotic pathway is disrupted, leading to the excessive accumulation of cells, tumorigenesis can be the result [25,64,65]. Therefore, our data suggest that the down-regulation of miR-125b after ALV-J infection may be closely related to tumor formation.

## 5. Conclusions

In summary, we identified the potential function of miR-125b during ALV-J infection in chickens. Our results show that the suppression of miR-125b reduces late-stage apoptosis in HP45 cells, which suggests that miR-125b functions as a tumor suppressor during ALV-J-induced tumorigenesis. Sema4D, the target of miR-125b, was found to be up-regulated following ALV-J infection, which reversed the effect of this miRNA on apoptosis. The regulation of apoptosis plays an important role in tumor development and formation. These findings provide new insights into the infection and tumorigenic mechanisms of ALV-J, specifically via the modulation of host miRNAs involved in tumor-associated cell progression.

## Figures and Tables

**Table 1:** Target genes involved in signaling pathways associated with cellular progression.

Pathway ID	Gene ID	Gene Name	KEGG and GO
ko: K16794	374224	Platelet activating factor acetylhydrolase 1b regulatory subunit 1 (PAFAH1B1)	Metabolic pathways Ether lipid metabolism
ko: K09208	427493	Krueppel-like factor 13 (KLF13)	Regulation of transcription from RNA polymerase II promoter
ko: K06521	396331	Semaphorin 4D/CD100 (Sema4D)	Axon guidance cell adhesion molecules
ko: no	445340	PR domain zinc finger protein 1 (PRDM1)	Regulation of transcription
ko: K03211	395750	ETS variant 6 (ETV6)	Transcriptional activator
ko: no	421301	Limb bud and heart development (LBH)	Regulation of stem cell differentiation regulation of transcription
ko: K11584	423460	Protein phosphatase 2 regulatory subunit B’gamma (PPP2R5C)	Cellular Processes mRNA surveillance pathway
ko: K04678	416487	SMAD specific E3 ubiquitin protein ligase 1 (SMURF1)	TGF-beta signaling pathway endocytosis
ko: K22040	420289	Transcriptional repressor GATA binding 1 (TRPS1)	Negative regulation of transcription
ko: K11850	424216	Ubiquitin specific peptidase 37 (USP37)	Ubiquitin system
